# A systematic review of neuroimaging and acute cannabis exposure in age-of-risk for psychosis

**DOI:** 10.1038/s41398-021-01295-w

**Published:** 2021-04-13

**Authors:** Lani Cupo, Eric Plitman, Elisa Guma, M. Mallar Chakravarty

**Affiliations:** 1 Integrated Program in Neuroscience, McGill University, Montreal, QC Canada; 2 Computational Brain Anatomy (CoBrA) Laboratory, Cerebral Imaging Center, Douglas Research Centre Verdun, Montreal, QC Canada; 3 Department of Psychiatry, McGill University, Montreal, QC Canada; 4 Department of Biological and Biomedical Engineering, McGill University, Montreal, QC Canada

**Keywords:** Pathogenesis, Neuroscience

## Abstract

Acute exposure to cannabis has been associated with an array of cognitive alterations, increased risk for neuropsychiatric illness, and other neuropsychiatric sequelae including the emergence of acute psychotic symptoms. However, the brain alterations associating cannabis use and these behavioral and clinical phenotypes remains disputed. To this end, neuroimaging can be a powerful technique to non-invasively study the impact of cannabis exposure on brain structure and function in both humans and animal models. While chronic exposure studies provide insight into how use may be related to long-term outcomes, acute exposure may reveal interesting information regarding the immediate impact of use and abuse on brain circuits. Understanding these alterations could reveal the connection with symptom dimensions in neuropsychiatric disorders and, more specifically with psychosis. The purpose of the present review is to: 1) provide an update on the findings of pharmacological neuroimaging studies examining the effects of administered cannabinoids and 2) focus the discussion on studies that examine the sensitive window for the emergence of psychosis. Current literature indicates that cannabis exposure has varied effects on the brain, with the principal compounds in cannabis (delta-9-tetrahydrocannabinol and cannabidiol) altering activity across different brain regions. Importantly, we also discovered critical gaps in the literature, particularly regarding sex-dependent responses and long-term effects of chronic exposure. Certain networks often characterized as dysregulated in psychosis, like the default mode network and limbic system, were also impacted by THC exposure, identifying areas of particular interest for future work investigating the potential relationship between the two.

## Introduction

In recent years there has been a surge in public policy decriminalizing or legalizing recreational cannabis use worldwide^1,2^. In spite of these changing norms, our understanding of the mental health consequences of cannabis exposure remain inconclusive. From a clinical standpoint, there is an emerging consensus on how cannabis may confer some therapeutic benefits (treatments for chronic pain and glaucoma)^3,4^, and may also increase risk for adverse mental health outcomes (major mental illnesses and associated symptomatology)^5^. Specifically, cannabis use has been associated with increased risk for depressive^6^ and anxiety disorders^7^, and, central to this review, psychosis spectrum disorders^8^. Cannabis use initiated during early adolescence confers the greatest risk for adult psychosis^9^, and dose-dependent cannabis use has been associated with an increased likelihood of developing psychosis and schizophrenia^8^ while short-term cannabis use has been associated with increases in psychotic-like symptoms, such as altered perception and anxiety^10^. Risk during adolescence could in part be conferred from critical periods of development in neurotransmitters. Development of the GABA-ergic (γ-aminobutyric acid) system during adolescence has been associated with response inhibition and working memory^11^. During the same time period, there occurs pruning of glutamatergic neurons, and reductions in innervation in the dopaminergic system during typical development^11^.

While cannabis contains many compounds responsible for various physiological effects, tetrahydrocannabinol (THC) is the psychoactive component most associated with psychotomimetic effects^12^. THC binds native cannabinoid receptors, such as G-protein coupled receptors like CB1, which acts as a receptor for endocannabinoids like anandamide^12^. CB1 receptors are distributed in various brain regions, and expressed on the presynaptic axon terminals of different types of neurons including GABAergic and glutamatergic neurons^13^. As an inhibitory neurotransmitter, active GABA-ergic synapses reduce the likelihood that postsynaptic neurons will fire. When THC or endocannabinoids bind to CB1, however, they prevent the release of GABA, permitting the postsynaptic cell to fire. An example of this prevention is dopamine, where GABA-ergic synapses control the release of dopamine into the system. Therefore, in the presence of THC, dopaminergic neurons are not prevented from firing, leading to an overabundance of dopamine. CB1 receptors are present in a high density in GABAergic axon terminals from the striatum^14^, potentially relating to excess dopamine in the striatum.

Increased dopamine in the striatum coincides with the dopamine hypothesis of schizophrenia as individuals with schizophrenia display excess levels of dopamine in the striatum, thought to be related to positive symptoms like hallucinations^15^. According to the dopamine hypothesis, patients with schizophrenia have reduced levels of dopamine in the prefrontal cortex (PFC) associated with cognitive impairments and negative symptoms like anhedonia^15^. The excitatory neurotransmitter, glutamate is additionally dysregulated in schizophrenia^16,17^, notable as glutamatergic synapses also express CB1 in the presynaptic cell. When THC binds CB1, less glutamate is released into the system, relevant to the effects seen in psychosis^17,18^.

In addition to THC, other compounds in cannabis, such as cannabidiol (CBD) have a host of differential pharmacological effects on the brain with demonstrably different impacts from THC. Like the endocannabinoid 2-Arachidonoylglycerol, CBD binds CB2, a receptor that has not been as well characterized as CB1 but is largely present in the immune system^19^. CBD has been posited to have neuroprotective effects, reducing the effects of THC^20^. Previous research also suggests that exposure to cannabis with a high THC concentration increases risk compared with low-potency cannabis^21^. Both THC content and THC:CBD ratio in recreational cannabis seized by California law enforcement increased significantly between 1996 and 2008^22^.

Psychoses generally emerge earlier for men (mean age of first episode: 24.2, mean age of first negative symptom: 26.5) than for women (mean age of first episode: 27.4, mean age of first negative symptom: 41.6)^23^. There is a higher incidence of schizophrenia among men (1.4:1); however, prevalence rates are similar, and women predominate at older onset^24^. Although the cause of the discrepancy is unknown, it has been suggested that sex hormones, such as estrogen and testosterone, may contribute to the sex differences^24^. Given that females are typically more sensitive to the effects of cannabis use as they relate to psychosis^25^, it is important to examine sex differences in cannabis response as a means to better understand this differential susceptibility

In this review, we examine studies that administer cannabinoids to better understand how mechanisms of acute exposure during adolescence and young adulthood may be implicated in changing of brain circuitry, thereby increasing risk for the emergence of psychoses. While understanding the impact of chronic use is critical, habituation makes it difficult to tease apart how cannabis alters specific brain circuits. Studies investigating chronic use are limited by confounding variables, such as concomitant tobacco^26^, alcohol^27^, and polydrug use^28^, as well as shared genetic risk for psychosis and cannabis use^29^. By focusing on acute studies, this review reduces the confounding effects associated with repeated cannabis use. To mitigate the chance that genetic background may increase psychosis-proneness and cannabis use, we examine studies that use neuroimaging techniques to investigate how brain circuits and behavioural responses are altered following acute cannabis exposure. The alterations may reflect underlying alterations to the GABAergic, glutamatergic, and dopaminergic systems that undergo refinement during adolescence^11^. To capture the state of cannabis research, this review includes THC, CBD, as well as homologues of these molecules, such as tetrahydrocannabivarin (THCv). We synthesize the neuroimaging studies in humans and animal models that examine the effects of cannabinoid administration, both cross-sectionally and longitudinally in an age group coincident with the typical age-of-onset of psychosis (20–22; however there are additional spikes reported around 40 for women, and even some accounts of a third spike for women around 80)^30,31^ to better understand the impact of cannabinoids on the brain during these sensitive periods^23^.

The translational neuroimaging focus of this review aims to demonstrate how whole-brain investigations of the effects of cannabis on brain function, the activity of specific receptor families, and neurochemistry can be contextualized across species. Ultimately this review seeks to reveal the state of understanding the effects of acute cannabis exposure and how this relates to the etiology of psychosis. We provide it as a reference for researchers planning projects to identify gaps in the literature and opportunities for further investigation.

## Methods

### Literature search

**Fig. 1:**
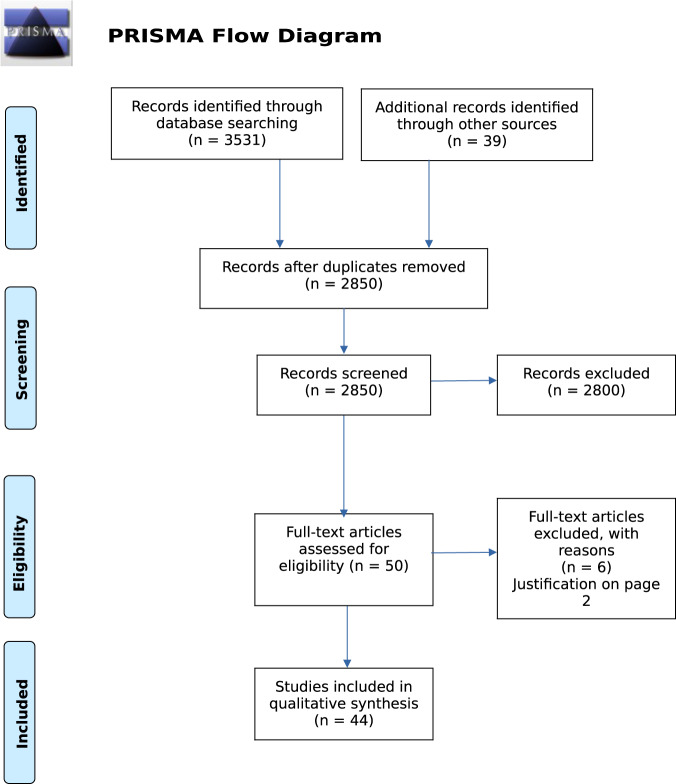
PRISMA flow diagram. PRISMA flowchart illustrating process of systematic review inclusion and explanation for excluded studies.

Neuroimaging techniques, such as functional magnetic resonance imaging (fMRI) and positron emission tomography (PET) are ideal for detecting the acute effects of cannabis exposure on brain function. Additionally, they permit translational approaches to research questions, including studies in both humans and non-human animals, the latter of which represents an opportunity for further research as few studies to date utilize neuroimaging techniques to study the effects of cannabinoids on non-human animal brains. We used this premise to guide our Ovid search of Medline, Embase, and PsycINFO (1980-June Week 2, 2019) to identify articles that used neuroimaging to assay brain function in populations within an age-range relevant to the development of psychosis-like symptoms (see below) and with acute exposure to cannabinoids. Search terms included: (magnetic resonance imaging or MRI or functional magnetic resonance imaging or fMRI or positron emission tomography or PET or diffusion tensor imaging or DTI or computed tomography or CT or magnetic resonance spectroscopy or MRS) and (cannab* or tetrahydrocannabinol or THC or marijuana) and (adolescen* or develop* or teenage* or matur* or youth or young). Additionally, reference sections of major relevant reviews^32–34^, were reviewed for applicable articles that were potentially missed. Included studies and reviewed articles are reflected in the PRISMA flow chart (Fig. 1).

### Inclusion criteria

Inclusion criteria were full-length, English-language articles that employed in vivo neuroimaging (using MRI, MRS, PET, CT, and DTI) in humans aged 14–40 (>90% of the sample) or adolescent aged non-human animals (mouse: postnatal day [PND] ~23–50^35^, rat: PND ~28–60)^36^ as well as administration of synthetic or natural cannabinoids.

### Exclusion criteria

Exclusion criteria for the systematic review included comorbid psychiatric disorders, administration of synthetic cannabinoid receptor agonists, or case-studies.

## Results

After deduplication, the Ovid search yielded 2811 results. All titles and abstracts were reviewed by L.C., and either E.G. or E.P. (each reviewed half). Forty-four articles (40 human and four preclinical studies) met the inclusion criteria and underwent full-text assessment for eligibility (Table 1). In the following section, we provide an overview of experimental methodology and summarize behavioral results before synthesizing neuroimaging findings across studies. In order to compare networks affected by cannabis exposure and those altered across the spectrum of psychosis, studies from clinical high risk (CHR), first episode psychosis (FEP), and schizophrenia are included at the end of results sections by modality where available.

**Table 1:** Study information and summary of detailed results.

Author (year)	Method	Species	Age: Mean (SD)	*N*(females)	Drug	Dose, Route	Multiple comparison corrections	Detailed results
Atakan^37^	e-r fMRI tasks: response inhibition	Human	26.76(5) range = 20–42	21(0)	THC	10 mg, Oral	NC	During no-go compared to oddball: THC increased activation in the HC, tail of the caudate nucleus, right In. THC increased activation in the right MTG in the transiently psychotic group and attenuated activation in the non-psychotic group
Barkus^66^	[123I]IBZM SPET	Human	26.3(4.2)	9(0)	Dronabinol	2.5 mg, IV	NC	No difference in striatal dopamine release
Battistella et al.^59^	e-r fMRI tasks: tracking	Human	24(3) range = 18–30	31(0)	Bedrobinol, 11% THC, <1% CBD	0.7 g CB, ~42 mg THC, inhaled	MCC	THC increased BOLD in a cluster covering the ACC and vmPFC. THC decreased BOLD in anterior In, dorsomedial Thal, left middle frontal gyrus. THC induced relative decrease in activation in anterior In, dorsomedial Thal, Stri, right dlPFC, right superior parietal lobule and cerebellum
Bhattacharyya et al.^67^	e-r fMRI tasks: verbal memory	Human	26.7	15(0)	THC and CBD	THC: 10 mg; CBD: 600 mg, Oral	NC	THC increased PANSS scores (negative and general subscales). THC augmented parahippocampal, cingulate, and PFC activation during the task. THC decreased activation in the bilateral striatum and rostroanterior cingulate
Bhattacharyya et al.^68^	e-r fMRI: tasks: verbal memory, response inhibition, sensory processing, fearful face viewing	Human	26.7(5.7)	MRI: 15(0) Behavior: 6(3)	THC and CBD	THC: 10 mg; CBD: 600 mg, Oral	MCC	Retrieval phase: THC and CBD had opposite effects in Stri, ACC, medial PFC, and lateral PFC. Effects of THC inversely correlated with severity of psychotic symptoms: THC attenuated Stri. Fearful faces: THC and CBD had opposite effects on activation in left Amyg, fusiform and lingual gyri, lateral PFC. THC augmented amygdalar response to fearful faces, correlated with levels of anxiety. CBD attenuated amygdalar response. Go/no-go task: opposite effects in parahippocampal gyrus bilaterally, left In and caudate: THC attenuated. Speech listening: opposite effects in lateral temporal cortex bilaterally. Checkerboard viewing: opposite effects in occipital cortex bilaterally
Bhattacharyya et al.^69^	e-r fMRI, tasks: attention	Human	26.7(5.7)	15(0)	THC and CBD	THC: 10 mg; CBD: 600 mg, Oral	MCC	THC increased activation in the right inferior, middle, and superior frontal gyri, right orbitofrontal cortex, frontal pole, attenuated activation in the head of the caudate, putamen, In, right thal
Bhattacharyya et al.^38^	e-r fMRI, tasks: response inhibition	Human	26.5(5.8)	36(0)	THC	10 mg, Oral	MCC	Response inhibition: THC attenuated activity in left inferior frontal gyrus and adjacent In, left precuneus. THC augmented right HC, caudate nucleus
Bhattacharyya et al.^74^	e-r fMRI tasks: verbal memory	Human	25.35 (5.24)	CBD: 16(6), placebo: 17(10), HC: 19(8)	CBD or vehicle	600 mg, Oral	MCC	Relative to PCBO, during encoding: CBD increased activity in left parahippocampal gyrus and reduced activity in precentral gyri. Relative to PCBO, during recall CBD: increased activation in left cingulate gyrus, right precentral gyrus, medial frontal gyrus. During encoding: clusters PCBO > CBD > CT: right inferior frontal and mid-frontal gyri and In, left In and putamen, precentral gyri, right fusiform gyrus, left cerebellum
Borgwardt et al.^70^	e-r fMRI tasks: verbal memory	Human	26.7(5.7), range = 20–42	15(0)	THC and CBD	THC: 10 mg; CBD: 600 mg, Oral	MCC	THC: no-go relative to oddball: activation in right HC, right postcentral gyrus, lingual gyrus bilaterally. CBD: activation in superior and middle temporal gyri and In bilaterally and in right posterior cingulate gyrus. Overall: THC reduced activation in right inferior frontal gyrus, ACC, bilateral precuneus. THC increased activation in right HC/parahippocampal gyrus, right superior and transverse temporal gyri, right fusiform gyrus, right caudate and Thal, left posterior cingulate and precuneus. CBD: reduced activation in left In and left superior and transverse temporal gyri
Bossong et al.^57^	[^11^C]-Raclopride PET	Human	21.9(2.7) range = 20–27	7(0)	THC	8 mg, vaporized	NC	THC reduced dopamine receptor availability in ventral Stri and precommissural dorsal putamen
Bossong et al.^39 a^	e-r fMRI tasks: working memory	Human	21.4(2.1) range = 18–27	17(0)	THC	6 mg, followed by 3 maintenance doses of 1 mg, vaporized	MCC	THC reduced load-dependent increase in activity associated with task. Linear interaction between drug and load. The harder the task, the more THC impacts activity. Significant linear difference in load between PCBO and THC in left dlPFC, left inferior temporal gyrus, left inferior parietal gyrus, and cerebellum
Bossong et al.^40 a^	e-r fMRI Tasks: associative memory	Human	21.6(2.1), range = 18–27	14(0)	THC	6 mg, followed by 3 maintenance doses of 1 mg, vaporized	MCC	During encoding: THC decreased activity in right In, right inferior frontal gyrus, left middle occipital gyrus. recall: THC increased activity in left and right precuneus
Bossong et al.^41^ ^a^	e-r fMRI tasks: continuous performance	Human	22.0(4.9), range = 18–40	23(0)	THC	6 mg, followed by 3 maintenance doses of 1 mg, vaporized	NC	Task-induced deactivation (TID) in ROIs: activity increased after THC. TID regions were more sensitive to the effects of THC than task-induced activation networks. After THC, negative correlation with TID activity and task performance
Bossong et al.^42^ ^a^	e-r fMRI tasks: emotional processing	Human	21.5(2.5) range = 18–26	11(0)	THC	6 mg, followed by 3 maintenance doses of 1 mg, vaporized	NC	THC had a different effect on happy and fearful face (FF) viewing. THC decreased activity in FF condition. Interaction between drug and condition in vermis, left occipital cortex, right occipital cortex, left HC, right PFC, right superior parietal gyrus, right sMA
Colizzi et al.^43^	e-r fMRI tasks: verbal memory, response inhibition	Human	26.0(5.6)	24(0)	THC	10 mg, Oral	MCC	THC induced greater activity in the left medial frontal gyrus and left inferior frontal gyrus. Decreased activity in left cingulate gyrus and in the culmen and cerebellar lingual bilaterally. Left medial frontal gyrus deactivated in nonusers (NUs)s in PCBO condition, but activated by NUs in THC and cannabis users (CU)s in PCBO. Parahippocampal gyrus deactivated in THC. Facial expressions: THC reduced activity in right inferior frontal and middle frontal gyrus, declive, uvula, fusiform gyrus. Left brain areas found interaction between drug and lifetime use: NUs in placebo activated left fusiform gyrus and deactivated left precuneus, cuneus, left posterior cingulate
Colizzi et al.^44^	e-r fMRI, tasks: attention, fearful face viewing	Human	26.0(5.6)	24(0)	THC	10 mg, Oral	NC	No significant effect of THC during encoding for verbal memory, but there was an interaction between drug and previous cannabis exposure: encoding + PCBO, activation in right superior temporal gyrus in NUs, encoding + THC activation here decreased in NUs. NUs: THC changed activation in left parahippocampal positively correlated with severity of psychotic symptoms. during response inhibition: THC increased activation in the right anterior cingulate and reduced it in left In. Involvement of left inferior parietal lobule during inhibition control, THC had different effects for cannabis users and NU
Colizzi et al.^45^	MRS	Human	24.4(4.29)	16(9)	THC	1.19 mg/2 ml, IV	NC	THC increased Glutamate+Glutamine (Glx) in the left caudate head, positive correlation between previous cannabis exposure and increase in Glx, Glx levels were lower in subjects who were sensitive to THC-induced psychotomimetic effects
Dalton and Zavitsanou^83^	PET: [^11^C]-Raclopride, [3H] SCH 23390	Rats (Wistar)	PND 35 or 70	54 PND 35, 45 PND 70	HU 210	25, 50, or 100 mg	NC	After 14 days of HU 210 in adults, dose-dependent increase in D1 receptors in lateral caudate putamen and olfactory tubercle. After single injection in PND 35, overall effect on D2 receptors
de Sousa Fernandes Perna et al.^46^	e-r fMRI, tasks: alcohol vs. cannabis marketing	Human	22.5(2.3)	62(26)	THC	300 mg/kg bodyweight in 2 doses	NC	Main effect of group in left HC and right precuneus. After intoxication (cannabis or alcohol), there was a main effect of marketing on BOLD response in postcentral cluster, cingulum, temporal, parietal, frontal, and occipital cortices. Main effect of intoxication on bold in right supplementary motor area (reduction)
Fusar-Poli et al.^71^	e-r fMRI, tasks: emotional processing	Human	26.67(5.7) range = 18–35	15(0)	THC and CBD	THC: 10 mg; CBD: 600 mg, Oral	NC	For 50% fearful faces, CBD decreased activation in a region in posterior lobe of cerebellum bilaterally. 100% fearful faces: CBD attenuated bold signal in left medial temporal region (Amyg) and anterior and posterior cingulate gyri, left middle occipital gyrus, right posterior lobe of cerebellum. Neutral faces: THC increased activation in posterior-middle temporal gyrus, left inferior parietal lobule. 50% fearful faces: THC increased activation in right inferior parietal lobule, but decreased activation in left medial frontal gyrus. 100% fearful faces: THC increased activation in left precuneus and in primary sensorimotor cortex bilaterally; decreased activation in middle frontal gyrus bilaterally and in posterior cingulate gyrus
Ginovart et al.^79^	PET: [^18^F]fallypride and 3H-(+)-PHNO	Rats(Sprague-Dawley)		4–15(0)	THC in saline/ethanol/cremophor	1 mg/kg/day IP	NC	THC increased binding potential of 18Ffallypride in dorsolateral Stri
Gorka et al.^61 b^	e-r fMRI, tasks: emotional processing	Human	20.8(2.6) range = 18–28	16(8)	Marinol in dextrose	7.5 mg, Oral	MCC	Altered functional coupling between left basolateral Amyg and rostral ACC/medial PFC as well as left superficial Amyg and rACC/mPFC. THC increased left basolateral Amyg to ACC/mPFC connectivity
Gorka et al.^62 c^	e-r fMRI, tasks: emotional processing	Human	25.43 (5.33)	78(44)	Marinol in dextrose	7.5 mg, Oral	NC	Group by instruction interaction in left Amyg. Within THC group, left Amyg activation increased during “maintain” compared with “look”. Group by condition interaction between both Amygdalae and dlPFC. Compared with PCBO, THC decreased Amyg-dlPFC coupling during reappraise and maintain, and during look, it increased left Amyg-dlPFC coupling
Higuera-Matas et al.^82^	PET: [^18^F]-FDG	Rats (Wistar)	P28–P38	Saline: 16(9), CP: 18(12)	CP 55, 940	0.4 mg/kg/day, IP	NC	Increased activation in frontal cortex in CP 55 females. No changes in males
Jansma et al.^47 a^	e-r fMRI: monetary incentive delay	Human	21.2(0.8) range = 18–26	21(0)	THC	6 mg + 1 mg/30 min, Vaporized	NC (2 ROIs)	NAcc during anticipation: After THC, lower response in nicotine addicts (NAD) than CT. CPu during anticipation: CT increase in CPu brain activity with increased reward. THC, smaller effect of reward in NAD than in CT
Klumpers et al.^48^	rs fMRI	Human	22.17(2.95) range = 18–45	12(3)	THC	3 doses, 2, 6, and 6 mg at 1.5 h intervals, vaporized	MCC	THC altered connectivity in sensorimotor, left and right dorsal visual stream networks. After THC, increases in right dorsal visual stream connection with left and bilateral frontal pole as well as dorsomedial PFC and left superior PFC. Connectivity decreased in right dorsal visual stream (superior frontal pole, middle and inferior frontal gyrus, dlPFC). Increase of connectivity found between cerebellum and sensorimotor network (occipital pole, lateral occipital cortex) and the dorsal visual stream network
Lee et al.^49^	e-r fMRI, tasks: pain response	Human	*R* = 24–34	12(0)	THC	15 mg, Oral	MCC	Interaction between capsaicin and THC in ACC: THC decreased activity in response to capsaicin. THC increased activity in right Amyg in response to noxious stimulation. Significant correlation between effect of THC on right Amyg (increase) and analgesic effect of THC. During pain state, THC reduced connectivity between right Amyg and primary sensory cortex
Mathew et al.^51 d^	SPECT: 133Xenon inhalation	Human	25.3(6.4)	20(0)	THC	3.55, 1.75, 0%, smoked	MCC	Cerebral blood flow increased following both low and high-doses of cannabis, especially in anterior regions of hemispheres. Changes in right hemisphere persisted longer
Mathew et al.^50 d^	SPECT:133Xenon inhalation	Human	21.7(8)	35(0)	THC	3.55, 1.75, 0%, smoked	NC	Drug by time interaction: increase of global cerebral blood flow following low and high cannabis doses, especially in anterior parts of each hemisphere
Nguyen et al.^81^	PET: [^18^F]-FDG	Rats(Wistar)	10–11 weeks of age	12(0)	HU-210 (*n* = 7) or vehicle (*n* = 5)	100 mg/kg, IP	NC	Interaction between time and treatment: HU-210 increased [^18^F]-FDG uptake on day 1
O’Leary et al.^52^	PET: [^15^O] water	Human	21.6(1.6)	12(6)	THC	20 mg, inhalation	MCC	In both groups, THC increased regional cerebral blood flow (rCBF) in anterior cingulate, mesial, and orbital frontal lobes, In, temporal poles, and cerebellum. THC reduced rCBF in auditory and visual cortices
O’Leary et al.^60^	e-r fMRI, tasks: sensory processing	Human	23.5(4.3)	12(6)	THC	20 mg, inhalation	MCC	THC increased regional blood flow in ventral forebrain: bilateral, orbital frontal lobe, anterior temporal lobe, In, subgenual anterior cingulate. THC increased blood flow in superior ACC, mesial frontal lobe, right and left cerebellar regions. THC decreased rCBF in mesial occipital lobe and precuneus. Additional interaction results
Phan et al.^63 b^	e-r fMRI, tasks: emotional processing	Human	20.8(2.6) range = 18–28	16(8)	Marinol in dextrose	7.5 mg, Oral	MCC	THC attenuated Amyg activation to threatening faces. No effect on primary visual and motor activation. Threat conditions: Right Amyg more activated in PCBO conditions than THC. THC increased Amyg activity in response to happy faces. Extent of attenuation of right Amyg activity related to extent of increase in “feel drug”(trend)
Rabinak et al.^64 b^	e-r fMRI, Tasks: Emotional processing	Human	*R* = 18–28	16(8)	Marinol in dextrose	7.5 mg, Oral	MCC	THC reduced subgenual ACC activity
Rabinak et al.^65 c^	rs fMRI	Human	25.43(5.05)	77(43)	Marinol in dextrose	7.5 mg, Oral	NC	THC associated with less static connectivity between Amyg and HC; greater dynamic connectivity between Amyg and vmPFC; low static connectivity between Amyg-HC after extinction learning associated with higher HC activation to conditioned stimulus during recall of extinction
Ramaekers et al.^53^	rs fMRI	Human	22.8(3.7)	122(26)	THC	450 mg/kg in two doses, 300 followed by 150, vaporized	MCC	Cannabis decreased functional connectivity between NAcc and left ACC, frontal lobe, left Thal,left Insula, temporal lobe, cerebellum, occipital lobe, In
Rzepa et al.^72^	rs fMRI	Human	*R* = 20–36	19(9)	THCv	10 mg, Oral	MCC	Left Amyg seed: THC reduced connectivity with the left precuneus and left posterior cingulate area (default mode network). Right dmPFC: increased connectivity with inferior frontal gyrus/medial frontal gyrus (dorsal visual stream)
Stokes et al.^88^	PET: [^11^C]-Raclopride	Human	33 (7)	13(6)	Marinol in dextrose	10 mg, Oral	MCC	Increase in psychotomimetic symptoms
Tudge et al.^73^	e-r fMRI, tasks: sensory processing	Human	25.4(4.5)	20(10)	THCv	10 mg, Unreported	MCC	THCv effect on chocolate sight: increased activation in putamen, ACC, caudate, mid-brain, cingulate gyrus. THCv effect on chocolate sight and taste: mid cingulate gyrus. Strawberry sight: In, mid orbital frontal cortex, superior temporal gyrus, putamen. Strawberry sight and taste: putamen, Amyg, In, mid orbital frontal cortex, superior temporal gyrus, Thal, caudate
van Hell et al.^54 a^	rs fMRI and ASL	Human	21.1(2.1) range = 18–27	26(0)	THC	6 mg, followed by 3 maintenance doses of 1 mg, vaporized	NC	Arterial spin labelling: THC increased perfusion in ACC, left superior frontal cortex, left and right In. Decreased perfusion in right post-central gyrus, left and right occipital gyri. Feeling high was negatively correlated with activity in superior frontal cortex and moderately positive with left anterior In. rs fMRI: THC reduced temporal signal to noise ratio in right In, left cerebellum, left substantia nigra
van Hell et al.^55 a^	e-r fMRI, tasks: reward processing	Human	21.7(2.3) range:18–27	11(0)	THC	6 mg, followed by 3 maintenance doses of 1 mg, vaporized	NC	THC during reward trials reduced reward-related brain activity. No ROI effects survived correction for multiple comparisons
Walter et al.^56 e^	e-r fMRI, tasks: sensory processing, pain response	Human	28(2.7)	15(7)	THC	10 mg, Oral	MCC	THC reduced activation in the right anterior In, HC, and cerebellum. THC decreased connectivity for ventral Thal and S2. THC influenced forward connections–THC decreased strength between Thal and S2, S2 and anterior In or HC
Walter et al.^58 e^	e-r fMRI, tasks: sensory processing	Human	26.6(2.9)	15(8)	THC	20 mg, Oral	MCC	THC reduced pleasantness of vanillin, correlated with reduced activation in the left Amyg, HC, and superior temporal pole
Winton-Brown et al.^86^	e-r fMRI, tasks: sensory processing	Human	26.7(5.7), range = 20–42	14(0)	THC and CBD	THC: 10 mg; CBD: 600 mg, Oral	MCC	Auditory stim: THC reduced activation in temporal cortex bilaterally in the anterior and posterior superior temporal gyrus and medial temporal gyrus and bilateral In, the supramarginal gyri, and in the right inferior frontal gyrus and left cerebellum. Correlation between reduction of activity in the right temporal cluster and increase in positive and negative symptom scale (PANSS) total. CBD increased activation in temporal cortex bilaterally, medially to the Insulae and caudally to the parahippocampal gyri and bilateral HC. CBD reduced activation relative to PCBO in a posterior-lateral region of the left superior temporal gyrus, incorporating parts of In, posterior middle temporal gyrus, and supramarginal gyrus. THCv CBD: CBD increased activation in right superior and middle temporal gyri. Visual stimuli: THC reduced activation in secondary visual cortex. Increased activation in right lingual and middle occipital gyri and in left hemisphere: increased activation anterior to lingual and fusiform gyri. Change correlated with increase in PANSS positive. CBD: increased activation relative to placebo in right occipital lobe. THCv CBD: THC augmented activation in left lingual and middle occipital gyri. THC attenuated activation in occipital regions bilaterally

Superscript letters indicate papers with overlapping samples.

*er fMRI* event-related fMRI, *HC* hippocampus, *In* Insula, *MTG* medial temporal gyrus, *STG* superior temporal gyrus, *ACC* anterior cingulate cortex, *vmPFC* ventromedial prefrontal cortex, *dlPFC* dorsolateral prefrontal cortex, *Thal* thalamus, *Stri* striatum, *PCBO* placebo, *CT* control, *sMA* supplementary motor area, *ROI* region of interest, *Glx* glutamate+glutamine, *NAcc* nucleus accumbens, *CPu* caudate putamen, *BOLD* blood oxygen level dependent signal, *PND* postnatal day.

### Human studies

The majority of human studies reviewed (*n* = 22) administered THC alone;^37–58^; methods of administration varied from vaporized (*n* = 11)^39–42,46–48,53–55,57^, to smoked (*n* = 4)^50–52,59,60^, and orally in gelatin capsules (*n* = 7; Table 1)^37,38,43,44,49,56,58^. The second most commonly administered cannabinoid was Dronabinol, a synthetic THC often prescribed medically and reported as Marinol (*n* = 6), administered orally [*n* = 5]^61–65^ and intravenously [*n* = 1])^66^. Studies that compared THC and CBD used gelatin capsules (*n* = 5)^67–71^. Remaining studies examined the THC homologue tetrahydrocannabivarin (*n* = 2)^72,73^, Bedrobinol (a strain of cannabis with 13.5% THC < 1% CBD) (*n* = 1)^59^, CBD alone (*n* = 1)^74^, or smoked cannabis without reporting CBD and THC concentrations (*n* = 1)^60^. This last study was the only one to include cannabis in its full form, while the others employed a dichotomy between THC and CBD. This work relates the human studies to relevant results from the psychosis spectrum literature (CHR [*n* = 3]^74–76^, first-episode psychosis [*n* = 1]^77^, and schizophrenia [*n* = 2]^76,78^).

### Preclinical models

All rodent studies administered the pharmacological intervention via intraperitoneal injection. These studies examined the effect of THC (1 mg/kg/day for 3 weeks)^79^ or CB1 receptor agonists Hebrew University 210 (HU 210)^80^ (single injection, 1 mL/kg)^81^, and CP 55,940 (PND 28–38, 2 mL/kg)^82^. Finally, one study examined the effects of acute and chronic HU 210 exposure on rats aged PND 35 and 70^83^. Both HU 210 and CP 55,940 have been demonstrated to be significantly more potent than THC, potentially limiting their comparison to cannabis use in humans^80,84^. One additional study in the search administered THC to Rhesus monkeys, however it falls outside of the inclusion criteria for age^85^.

### Imaging modalities

The majority of human studies used fMRI to investigate the acute effects of cannabis exposure using resting-state fMRI (rs fMRI; *n* = 5)^48,53,54,65,72^ or event-related fMRI (er fMRI; *n* = 27)^37–44,46,47,49,55,56,58,59,61–64,67–71,73,74,86^, (see Table 1 for classification by task-type). Arterial spin labeling (ASL; *n* = 1)^55^ and MRS (*n* = 1)^45^ were also used. Radioligand studies included PET and single-photon emission tomography (SPET/SPECT) (*n* = 6)^66^ (see Table 1 for summary of tracers).

The three rat studies used PET to examine either glucose metabolism using [^18^F]-2-fluoro-deoxyglucose ([^18^F]-FDG) (*n* = 2)^81,82,87^ or dopamine receptor activity with [^18^F]-Fallypride^79^. No preclinical studies used fMRI, ASL, or 1H-MRS.

### Behavioral results

Twenty-three studies reported the impact of cannabis on behavioral and psychometric assays in humans.

*THC studies*. The Visual Analogue Mood Scale (VAMS) was commonly used to index experiences related to “highness”/”being high”, “alertness”, “external perception”, “internal perception”, “contentedness”, and “calmness” to verify the effects of THC administration^39–44,50,52,54,55,59,63,64,71^. Rated with VAMS, THC exposure increased “drowsiness”, “nausea”, and “euphoria”^56,58^, but it reduced “alertness”^39,40,55^, “contentedness”^40,47^, “tranquility”^37^, and “calmness”^41,42^.

THC administration also increased reports of anxiety^37,43–45,48,50,71^, internal and external perception^40–42,47,48^, tension and anger^51^, sedation^43,45,71^, and confusion^59^. Assessments also revealed increased psychotic symptoms on the three Positive and Negative Syndrome Scale subscales (positive, negative, and general psychopathology)^37,43–45,69,71,88^.

*Comparison of THC and CBD administration*. There was evidence for increased intoxication, anxiety, sedation, and psychotic symptoms over time in response to THC, but not to CBD^70,86^. Additionally, one study with a small sample (six participants) reported that three of their participants experienced acute psychotic symptoms after THC, but these symptoms were ameliorated by pre-treatment with CBD^68^. Interpretation of the results of CBD exposure should be considered in the context of small, homogenous participant samples.

Taken together, these studies provide evidence that THC increases psychotic symptoms, anxiety, confusion, and sedation, while simultaneously reducing alertness, calmness, and contentedness. By contrast, CBD may be protective against these behavioral features.

### Biometric results

Studies examining biometric effects of acute cannabis exposure observed that THC exposure increased heart rate^39,40,45,48,52,54,55^ and blood pressure^41,42^. Further, reports of increased cortisol levels complement self-reports of increased levels of anxiety and tension^48^. Meanwhile, prolactin levels were reduced, possibly related to increased dopamine activity^48,89^.

### Neuroimaging studies

**Fig. 2:**
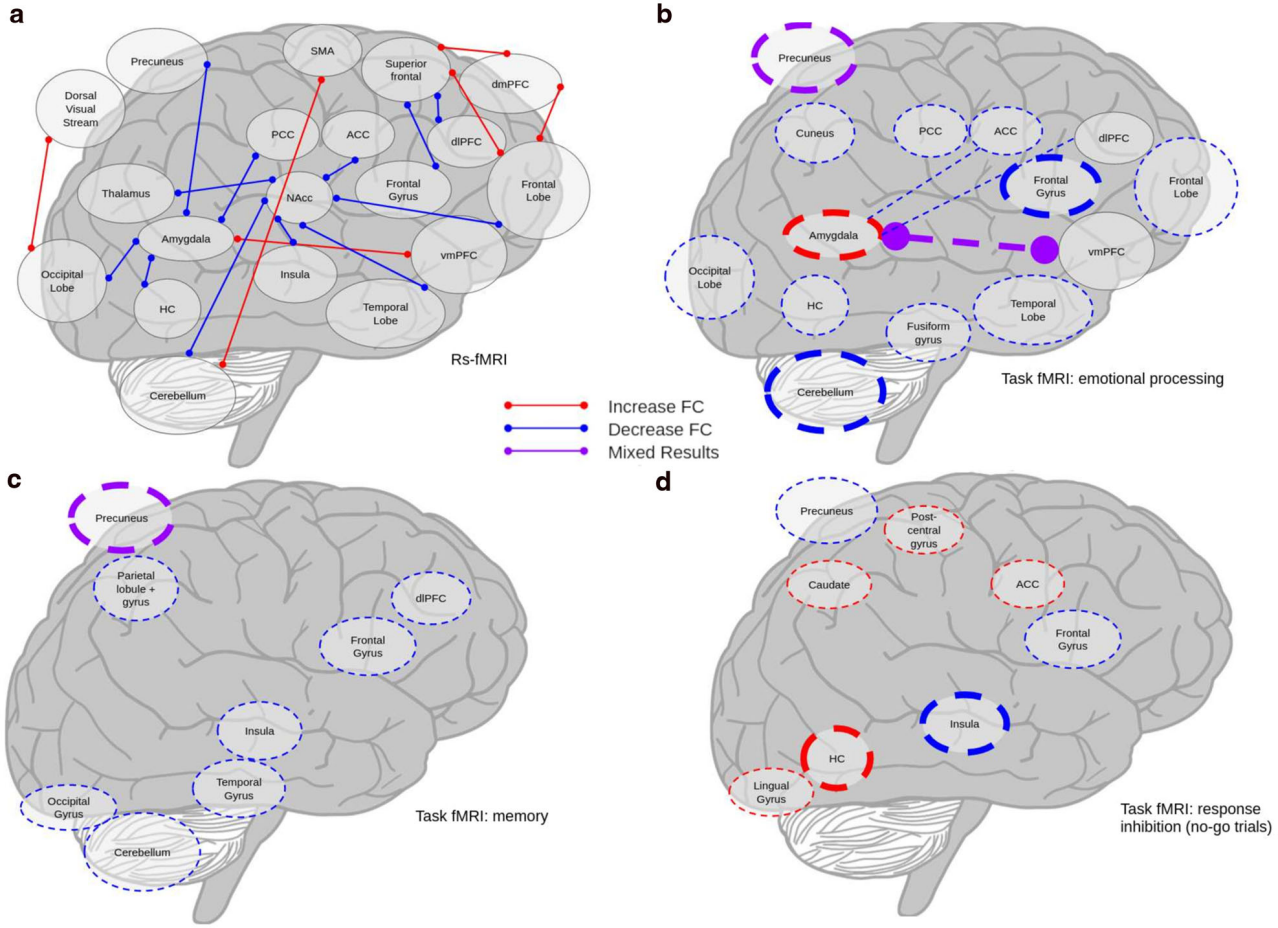
Visualization of main fMRI results across studies. **a** rs fMRI results, **b** er fMRI results, emotional processing tasks, **c** er fMRI results, memory tasks, **d** er fMRI results, no-go trials from response inhibition. Thin lines indicate results from one study. Thick lines indicate results from two. Solid lines indicate rs fMRI, dashed indicate resting state. Colored circles demarcate “activity” lines indicate “connectivity”.

**Fig. 3:**
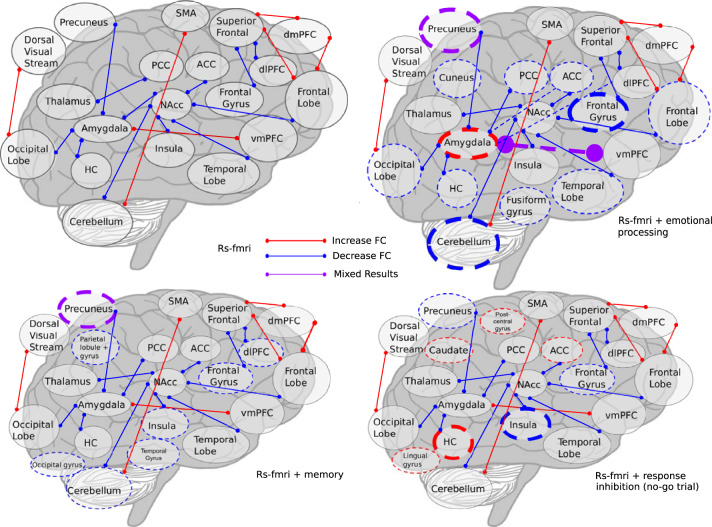
Visualization of fMRI results with er fMRI superimposed on rs fMRI. **a** rs fMRI results, **b** rs and er fMRI results; emotional processing tasks, **c** rs and er fMRI results; memory tasks, **d** rs and er fMRI results, no-go trials from response inhibition. Thin lines indicate results from one study. Thick lines indicate results from two. Solid lines indicate rs fMRI, dashed indicate resting state. Colored circles demarcate “activity” lines indicate “connectivity”.

**Fig. 4:**
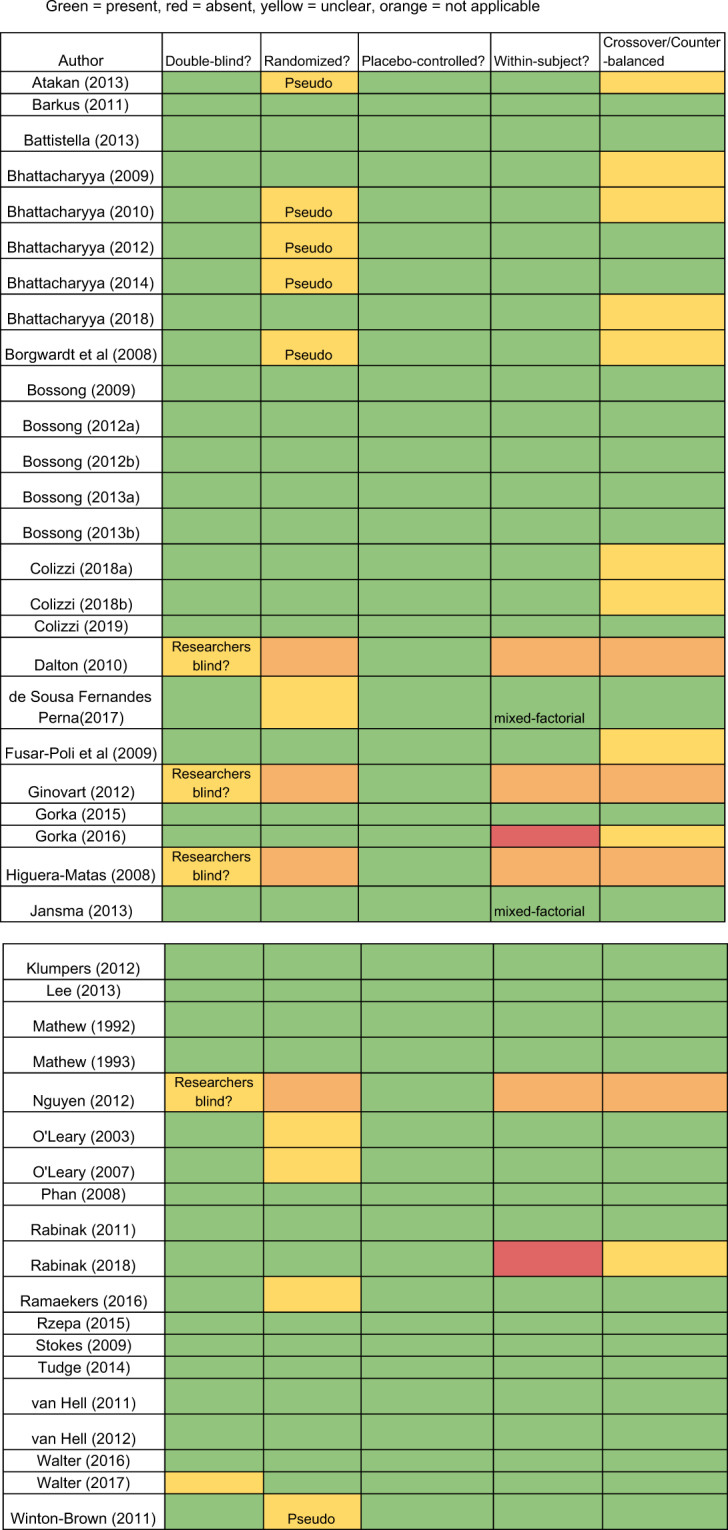
Risk of bias. Assesses likelihood of bias in each paper examining for double-blind, randomized, placebo-controlled, within-subject, and crossover/counter-balanced. Green = present, red = absent, yellow = unclear, orange = not applicable.

First, we report PET, rs and er fMRI, ASL, and MRS studies in humans; we further organize er fMRI studies by task type: emotional processing, memory, response inhibition, and sensory processing and examine those that do not cleanly fit into these categories. The final section investigates the preclinical studies together. Figure 2 provides a visualization of results from rs fMRI and key er fMRI studies following THC administration. Figure 3 provides a comparison with the er fMRI studies superimposed on the rs fMRI study results. Figure 4 provides a visual representation of Risk of Bias.

#### Radioligand studies

Three studies employed PET to examine striatal dopamine receptor availability^57^ and regional cerebral blood flow^52,60^. Additionally, SPET was used to examine dopamine release in the striatum^66^. One study also combined data from two previously published studies, and since both of the prior studies were included^57,88^, the third was excluded.

Eight milligram of vaporized THC reduced the binding potential of [^11^C]raclopride in the functionally limbic part of the ventral striatum^57^. However, in another study 10 mg did not alter binding of [^11^C]raclopride in the striatum^88^.

Twenty milligrams inhaled THC increased regional cerebral blood flow (rCBF) measured with [^15^O] water PET in cortical regions, and the cerebellum (see Table 1) and decreased rCBF in auditory and visual cortices^52^.

One study administered a single dose of 2.5 mg THC via intravenous injection and compared uptake of the tracer 123I-iodobenzamide in the basal ganglia. Following THC exposure, scores in the striatum ranged from a decrease by 16% to an increase by 34% and no results were significant, even though the dosages were large enough to elicit psychotic symptoms^66^.

*Radioligand studies in psychosis*. Increased striatal dopamine synthesis assessed with PET was associated with transition from prodrome to FEP in human participants^90^. Additional research suggests higher baseline striatal dopamine levels in patients with schizophrenia than healthy controls^91^. Following amphetamine administration, there is increased dopamine release in participants with psychosis than healthy controls^92^. These findings are in accordance with results suggesting THC exposure may increase striatal dopamine release^57^.

#### Resting-state fMRI

Five studies assessing rs fMRI observed divergent findings. See Table 1 for specific regions.

*Reward pathways*. A study examined the effects of 450 mg/kg vaporized THC on impulse control in cannabis users with bilateral nucleus accumbens seeds^53^. Cannabis decreased resting state functional connectivity (rs fc) between the accumbens and left anterior cingulate cortex (ACC), cortex, thalamus, and cerebellum.

*Fronto-Limbic pathways*. The impact of 10 mg THCv exposure was examined using a seed in the left amygdala^72^. Decreased connectivity with important “hub” regions such as the left precuneus and left posterior cingulate (key-default mode network [DMN] regions) was observed. THCv increased connectivity between a seed in the right dorsomedial PFC and the inferior frontal/medial frontal gyrus.

One study orally-administering 7.5 mg Marinol used specific regions of interest (ROIs: the amygdala, hippocampus [HC], and ventromedial PFC [vmPFC]) correlations to examine static and dynamic rs fc^65^. Their results indicated decreased static rs fc between the amygdala and HC, but increased dynamic rs fc between the amygdala and vmPFC.

*Whole brain analysis*. Using networks of interest^48^ and a voxel-wise technique^48,54^, rs fc was most altered in the right dorsal visual stream network following administration of 14 mg vaporized THC^48^. Increased connectivity with this region was localized in the frontal lobe. In the right hemisphere, THC decreased rs fc in the right hemisphere in other regions in the frontal lobe. Finally, THC increased rs fc between the cerebellum and sensorimotor network, and between the left dorsal visual stream and the occipital cortex. The second study reported the results of nine cumulative mg THC on temporal signal-to-noise ratio (tSNR; calculated by dividing mean blood-oxygen level dependent [BOLD] signal by its standard deviation over a time period; a measure thought to reflect greater spontaneous fluctuations and brain activity)^54^. THC reduced tSNR, in the right insula, left cerebellum, and substantia nigra, as hypothesized by the authors^54^. It is critical to note that results between the whole brain studies were markedly different, potentially due in part to the analytical techniques employed.

*rs fMRI in psychosis*. Rs fMRI studies in participants with a FEP reveal reduced connectivity in the DMN (dorsomedial PFC and posterior cingulate cortex (PCC)/precuneus) as well as weaker negative correlations between the lateral temporal cortex and the medial occipital lobe^77^. In patients with chronic schizophrenia, functional connectivity exhibits similar patterns, with decreased strengths of connectivity in the PFC, insula, and precuneus^93^.

The dorsomedial PFC was implicated in both THC exposure, where increased connectivity was observed with several regions^48,72^, and psychosis, where decreased connectivity was observed^77,93^. Both THC exposure and psychosis decreased connectivity in the precuneus^72,77,93^, as well as the occipital lobe^53,77^, insula^53,93^. While this may indicate regions for future investigation, the variability in results may also reflect statistical noise.

#### Event-related fMRI

Event-related fMRI experiments used emotional processing, memory, sensory perception, and response inhibition tasks (Table 1).

##### Emotional processing tasks

The amygdala is well-studied in the context of both THC exposure and emotional processing. A series of three studies assessed the effects of 7.5 mg orally-administered Marinol on emotional processing in sixteen participants^61–64^ and found that THC attenuated amygdala activation when viewing threatening faces^63^. The second study investigated rs fc between amygdala subfields and the cortex, revealing THC increased connectivity between both the amygdala and rostral ACC/medial PFC^62^, but was limited to viewing threatening faces. These findings suggest that the connection between these two regions may be especially integral to social threat processing and that THC exposure increases this connection, of special interest as previous research associates perception of social threat and symptoms of paranoia^94^. The final study examined limbic circuitry (amygdala and ACC) engagement in response to differing valence of stimuli, and observed that THC exposure reduced activity in the subgenual ACC and did not impact amygdala activity^64^. These results support the view that THC decreases activity in the limbic circuit; however, the lack of effect in the amygdala provides a point of contrast to the authors’ previous findings, which raises significant concerns about reproducibility and replicability.

In another task, participants were required to imagine positive contexts for negative images (e.g., reimagining a woman crying outside of a church as attending her wedding; a cognitive reappraisal task)^61^. An increase in left amygdala activity and decrease in bilateral amygdala-dorsolateral PFC coupling was observed during the reappraisal condition following THC administration (7.5 mg) compared with placebo. When matching emotional faces, 9 mg vaporized THC decreased activity during the fearful face condition in the cerebellum. While the decrease in activity during negative-expression-viewing is consistent with previous studies, the affected areas are inconsistent^62–64^.

To examine the impact of long-term cannabis use on emotional processing, one study examined fear processing in cannabis-users and nonusers (<5 exposures)^43^. In-study administration of 10 mg THC reduced activity in the right inferior frontal and middle frontal gyri, medial cerebellum, and fusiform gyrus. Cannabis users had greater activity in the right cingulate gyrus and left inferior parietal lobule. These findings further support that THC reduces activity, though once again identifying some novel areas of interest (such as the cuneus), while replicating others (such as the cerebellum).

Finally, two publications from the same study population and experiment examined the differential effects of THC and CBD on emotional processing^68,71^. When viewing fearful faces compared with neutral faces, 600 mg CBD reduced BOLD response in the left amygdala, left ACC, right PCC, and right cerebellum^71^. Ten milligram THC exposure during fearful face viewing increased activation in the left precuneus, but decreased it in frontal and temporal regions. During fearful face viewing, THC and CBD had opposite effects, with THC and placebo increasing amygdalar activation while CBD decreased it^68^. The authors also reported opposite effects in the fusiform and lingual gyri, lateral PFC, and cerebellum without specifying the directions of effects. Without more diverse samples, it is impossible to conclusively determine THC and CBD have opposite effects. Additionally, the reported results are not identical, necessitating further clarification of both methodology and the findings themselves. Visualization of the effects of THC administration during emotional processing tasks is presented in Fig. 2b.

*Emotional processing in psychosis*. Participants at risk for psychosis demonstrated altered activation in response to valenced faces when compared to control groups^75^. Unlike controls, the high-risk group showed a relative increase in activation in response to neutral rather than sad faces in the amygdala-hippocampal complex, thalamus, and cuneus. The amygdala^61–64,68,71^ and cuneus^43^ were implicated in emotional processing during cannabis exposure as well. Negatively valenced stimuli did not elicit as strong of a response in individuals with psychosis compared to neutral faces, the directionality consistent with response to CBD^68^, but not THC^62,68^.

##### Memory tasks

Previous evidence suggests chronic cannabis use can impair memory^95^. Six studies investigated the impact of THC on memory^39,40,44,67,68,74^.

*Verbal memory*. One study demonstrated that cannabis users and nonuser controls both during 10 mg orally-administered THC and placebo, deactivated the right superior temporal gyrus during the task^44^.

Another study found that following 10 mg THC administration, recall was associated with increased activity in the left dorsal ACC and medial PFC and decreased activity in the bilateral striatum and left rostral anterior cingulate gyrus, but found no influence of the administration of 600 mg of CBD^67^. Contradictory results are published in another study reporting on the same experiment in the same participant group, where the authors reported that THC and CBD had opposite effects in the striatum, ACC, and medial and lateral PFC during retrieval, with THC decreasing activity and CBD increasing it^68^. The same group also studied individuals at CHR for psychosis and found that 600 mg CBD decreased activation in the left parahippocampal gyrus during recall, but increased activation in the left cingulate gyrus, right precentral gyrus, and medial frontal gyrus^74^. There was a step-wise difference in activation across the three groups with the CHR group in the middle. These results provide intriguing evidence that CBD may normalize memory-task impairment for CHR populations.

*Additional memory tasks*. Two additional studies conducted with the same participants used the Sternberg item recognition paradigm^40^ and a pictorial memory task^39^. Difficulty of the Sternberg task can be scaled to allow for assessment of load-dependent increases in brain activity. Nine milligram of THC reduced load-dependent activity in the cortex and cerebellum^40^. In the pictorial memory task, THC reduced activity in the right insula, right inferior frontal gyrus, left middle occipital gyrus during encoding of images, and increased activity in the precuneus bilaterally during recall^39^. While the results differed in areas impacted by THC, both studies indicate that during encoding, THC reduces activity. Differing areas of impact could be due to the respective brain-areas employed in the tasks, however without replication it is also possible that the reported results reflect properties of the methodology, rather than the drug or task. Visualization of the impact of THC on memory tasks is provided in Fig. 2c.

*Memory tasks in psychosis*. In a verbal memory task, during encoding, participants at risk for psychosis showed decreased activation in the frontal and parahippocampal gyri compared to healthy controls^74^. Surprisingly, these results align with findings that CBD decreased activation in the parahippocampal gyrus during recall in participants at CHR for psychosis^74^.

##### Response inhibition tasks

Response inhibition was operationalized in a go/no-go test paradigm. In the no-go trials, 10 mg THC administration attenuated activation in the left inferior frontal gyrus, adjacent insula, and precuneus, which were all activated following placebo administration^38^ conversely THC increased engagement from the right hippocampus and caudate nucleus.

One study examined the impact of previous cannabis use on response to acute exposure during response inhibition^44^. Ten milligram THC increased activation in the right ACC and, similar to the above study, reduced activation in the left insula.

In a study examining the contrasting effects of 10 mg THC and 600 mg CBD, no-go trials following THC exposure were associated with greater activation in the right hippocampus, right postcentral gyrus, and bilateral lingual gyrus^70^. No-go trials in the CBD condition were associated with greater activation in the temporal gyri, insula, and PCC. While the drugs had distinct effects, they did not exhibit the same oppositional pattern present in the emotional processing studies. The findings of the go/no-go task employed in the aforementioned THC and CBD experiment were reported again in a paper highlighting the different effects of THC and CBD^68^. The authors reported finding opposite effects during the go/no-go in the bilateral parahippocampal gyrus, left insula, and caudate, with THC reducing activation and CBD increasing it. While the methods are reported as the same, the results differ between papers. The latter^68^ claims CBD and THC have opposite effects, while activation was varied in the initial paper^70^. Visualization of the impact of THC exposure on no-go trials is provided in Fig. 2d.

*Response inhibition in psychosis*. Comparing healthy controls to participants at CHR for psychosis and early schizophrenia during a go/no-go task, the right inferior frontal gyrus and bilateral dorsal ACC showed decreased activation during no-go relative to go in comparison with healthy controls, this pattern arising primarily from reduced no-go response activity^76^. THC also attenuated activation during no-go in the inferior frontal gyrus^38^, but increased activity in the ACC^44^.

##### Sensory processing

Five studies examined the effects of cannabis on sensory perceptions, examining gustation^73^, visual and auditory stimuli^68,86^, and pain^49,56^.

*Gustation*. The sole study examined how THCv impacted appetite depending on pleasant or aversive flavor and visual stimuli^73^. While 10 mg THCv did not change subjective stimuli ratings, it increased activity in response to the chocolate stimuli (paired visual and taste) in the caudate, midbrain, and cingulate gyrus. In response to a picture of moldy strawberries, THCv increased activation in the insula, frontal cortex, temporal gyrus, and putamen.

*Audition*. A study involving listening to neutral words read aloud demonstrated that THC reduced activity primarily in the temporal cortex, whereas CBD increased activity in the same region^86^. CBD also increased activity in the temporal gyri relative to THC. These results were replicated in a paper discussing the opposing effects of THC and CBD, where authors observe opposite directions of activation in the bilateral lateral temporal cortex^68^.

*Vision*. The same study investigating audition also examined the effects of cannabinoids on visual processing of checkerboard stimuli^68,86^. Relative to placebo, 10 mg THC reduced activity in the secondary visual cortex, and increased activity in the lingual, occipital, and fusiform gyri whereas 600 mg CBD increased activation in the right occipital lobe. THC increased activity in the left lingual and middle occipital gyri, also decreasing it in scattered areas of the occipital cortex and cerebellum relative to CBD. The opposite results in the occipital lobe are also reported in the larger study comparing THC and CBD activation^68^.

*Pain perception*. Two studies examined the effect of THC on pain perception supporting the use of cannabis as an analgesic^49,56^. One study demonstrated that 10 mg THC reduced activation in the right anterior insula, hippocampus, and cerebellum after inducing pain by activating trigeminal nociceptors with CO_2_^56^. An ROI analysis further revealed that THC decreased connectivity between the thalamus and secondary somatosensory cortex, which agreed with lower ratings of pain perception following THC exposure.

Fifteen microgram of THC decreased activity in the ACC in response to a topical application of capsaicin and lowered pain perception, but increased activity in the right amygdala in response to painful stimuli was correlated with the analgesic effects^49^. THC also reduced functional connectivity between the right amygdala and the primary sensorimotor cortex (S1) during ongoing pain, and decreased both subjective ratings of pain and limbic activity in response to painful stimuli.

*Pain perception in psychosis*. Patients with schizophrenia demonstrate reduced pain perception in comparison with healthy control, along with increased BOLD response in S1, but relatively reduced responsivity in the PCC, insula, and brainstem^78^. The analgesia reported in psychosis corresponds with that reported following cannabis exposure, as did reports of reduced activity in the insula^56^, however unlike individuals with psychosis, THC exposure decreased activity in S1^49^.

##### Remaining tasks

The remaining studies examined the effects of THC on monetary incentive delay^47,55^, cannabis marketing^46^, executive functioning^41^, attention^43,69^, and visuo-motor tracking^59^.

*Monetary incentive delay* (reward processing^55^). Nine microgram of THC reduced reward-related activity in the parietal cortex and temporal gyrus. These results indicate THC reduces responsivity to reward anticipation and presentation.

*Marketing*. THC (300 mg/kg) reduced BOLD signal in the right supplementary motor area in response to cannabis marketing^46^. Additionally, THC treatment overall reduced BOLD in the bilateral pallidum, striatum, and right caudate.

*Executive functioning*. Task-induced deactivation in a continuous performance task with identical pairs was observed in a network comprising the cortical regions and the cerebellum, which was more sensitive to the effects of 9 mg THC than other networks^41^. These findings indicate THC may dysregulate the DMN by increasing activity during tasks.

*Visual oddball detection*. Two studies used the visual oddball detection task, where participants respond to presentation of visual stimuli, to assess attention^69^. Relative to placebo, 10 mg THC increased activity in the right frontal gyri and frontal pole; THC also decreased activity in the right subcortical areas. CBD (600 mg) reduced activity in the left medial PFC and increased activity in similar subcortical areas. The second study examined the impact of previous cannabis use and found that after 10 mg THC exposure ingested orally, nonusers activated the left medial frontal gyrus, as did cannabis users after placebo^43^. Cannabis users in the THC condition deactivated the same area, as did nonusers in the placebo condition.

*Motor control*. One study examined the impact of 42 mg inhaled THC on psychomotor control with a visuo-motor tracking test to assess the impact of THC exposure on driving ability^59^. THC increased BOLD response in the ACC and ventromedial PFC, however it decreased activity in the thalamus and cortical regions. Combined with results that indicate impaired tracking of the target in the task, these findings shed light on the urgent need for more research of the effects of cannabis on psychomotor activity in relation to safe driving.

#### Arterial spin labeling

Examining ASL, 9 mg THC increased perfusion compared to placebo in the ACC, left superior frontal cortex and bilateral insula, and decreased perfusion in the postcentral and occipital gyri^54^. The increased perfusion associated with THC exposure may be explained by the vasodilative effects of cannabis.

#### Magnetic resonance spectroscopy

Ten milligram of orally-ingested THC increased rates of Glx (a pseudo-concentration of glutamate and glutamine) in the left caudate head, with the highest rates of increase in those who had the lowest levels of Glx in the placebo condition.

*MRS in psychosis*. Increased levels of glutamate in the dorsal caudate predicted transition to psychosis in CHR groups, and compared to healthy controls and those who did not transition, the transition group displayed higher rates of glutamate^96^. These findings correspond to increased rates of Glx following THC exposure^45^.

#### Animal models

Only four animal studies (all using PET) met the inclusion criteria. Radioactive tracers and rat background strains are listed in Table 1.

Nguyen et al. performed [^18^F]-FDG PET 15 min and 24 h following injection of 100 mg/kg HU 210 (a THC homologue) in 10–11-week old rats. They observed that HU 210 increased global uptake of [^18^F]-FDG only at the first timepoint, suggesting whole-brain hypermetabolism was acute and not persistent^81^.

Ginovart et al. administered daily 1 mg/kg THC injections for three weeks to male rats. While age was not reported, the reported weights of rats suggest that they were between 8 and 9 weeks old^97^. Results of the in vivo PET imaging revealed that THC increased D2 and D3 receptor availability in the dorsal striatum based on [^18^F]fallypride binding. Ex vivo autoradiography confirmed these findings, but also demonstrated increases in binding in the subcortical regions^79^.

Finally, after a single injection in PND 35, there was an overall effect of HU 210 on D2 receptors, however there was no interaction in individual regions^83^.

## Discussion

### Summary and implications

A systematic review of the literature investigating cannabis administration and neuroimaging reveals the heterogeneity in both methodology and findings. Overall, in rs fMRI, certain findings converge, despite differing analytical approaches. After the administration of both THC and THCv, there is increased connectivity between the dorsomedial PFC and the dorsal visual stream network both in the seed-based and whole-brain approach^48,72^. In order to facilitate interpretation and comparison with previous studies, future rs fMRI work should utilize multiple techniques for analysis, such as whole-brain voxel-wise analyses, seed-based approaches, and predefined ROIs, to examine in a single population which findings consistently appear across methodologies.

Event-related fMRI studies show disappointingly divergent results, for example THC both increases and decreases BOLD response in the amygdala during negatively valenced emotional stimuli^61,63^. Experimental design may change the effects of THC on pain sensitivity, with THC generally decreasing activity, but in different regions^49,56^. Small sample sizes and the absence of replication among studies limit the generalizability of results. The limited agreement among studies is illustrated in Figs. 2 and 3. In part, the lack of agreement could be due to focused analyses, such as the emphasis on the nucleus accumbens, which one study identified as a seed region^53^, whereas this area is not significant in studies performing whole-brain analyses. Figure 3 further demonstrates the lack of coherence among studies, examining the concurrence between rs and er fMRI studies. The diversity of results renders it difficult to draw meaningful conclusions across studies, but ultimately highlights the need for more rigorous research into the effects of cannabinoids. Given the well publicized issues with underpowered task and rs fMRI studies^98,99^, investigating the acute impact of cannabis exposure will require that studies be designed to be generalizable (large samples of diverse individuals, multiple-sites, and harmonized whole-brain analyses), supporting robust conclusions.

Preclinical studies represent a major opportunity for future studies as cannabis or THC can be administered experimentally either one or many times to study either short-term or chronic effects. Neuroimaging and behavior can be assessed at multiple time points and supplemented with post-mortem assays to develop a deeper characterization of the effects of cannabis exposure. As no rodent studies utilized fMRI, ASL, or 1H-MRS, they represent areas of special interest, even acknowledging challenges such as the confounding effects of anaesthesia regimens^100^ and obtaining high signal-to-noise ratio^101^. Additionally, while the preclinical studies administered cannabinoids through injections, most human studies administered it orally. Intravenous THC exposure mimics exposure by smoking, however following oral consumption, THC is first metabolized by the liver, reducing bioavailability^102^. Differences in method of exposure could limit the comparability between human and preclinical studies. This too presents a limitation to synthesis between human results, as there is heterogeneity in methods of exposure.

### fMRI limitations

The majority of studies included in this review examined either rs or er fMRI, however limitations, both inherent to this methodology and in terms of study design, impose limitations on the synthesis of results, such as the small sample sizes. Only four fMRI studies include more than sixty participants. Small sample sizes run the risk of being under-powered, leading to greater numbers of false negatives and overestimated effect sizes^103^. Future research should include power analyses and adequate sample sizes to further verify early findings in the field.

### THC and psychosis

A major focus of this review is the potential relationship between THC exposure and psychotic symptoms/schizophrenia. Not only does chronic cannabis use increase the risk of developing psychosis^104^, but also reviewed studies demonstrate acute cannabis exposure increases temporary psychotomimetic symptoms^37,43–45,69,71^. There is also convergence between fMRI studies in FEP and the effects of acute THC exposure, such as decreased activity in the dorsolateral PFC^48,105^. Additionally, most alterations were focused in the PFC and limbic areas, similar to seven other studies in this review^39,48,53,59,61,62,68,105^. Similar patterns of disrupted activity are seen between both pharmacological intervention with THC and in populations with FEP complementing symptomatic similarities, such as PANSS scores. Given areas of correspondence between THC administration and psychosis, future studies seeking a mechanistic connection between THC exposure and the emergence of psychosis should consider investigating the DMN (including the medial PFC, PCC, and inferior parietal lobules)^40,41,43,55,62,65,67,69,72,106,107^. The limbic system, comprising the cingulate cortex, parahippocampal region, hippocampus, and amygdala, was also highly impacted by THC and psychosis meriting further investigation^42,43,49,53,60–63,65,67–69,72–74,77,107^.

### Sex

Only 17 of the 39 reviewed human studies included female participants^45,46,48,52,53,56,58,60–65,68,72,74,82^; similarly only one of four non-human animal experiments included female rodents^82^. One of the groups that used the same sample for seven studies included in this review^39–42,47,54,55^ attributed their choice of recruiting only males to the “expected interactions between hormonal cycle and brain activity patterns in women, which will flaw the design. In addition, there is evidence for sex differences in the effects of THC”, citing a review of behavioral studies demonstrating sex-differences in adult rodents^108^. We hope that future researchers no longer cite the mysteries of having to deal with “female hormones” as an excuse for incomplete study design. Given the number of studies that adopt this philosophy, there is an urgent need for pharmacological studies involving females^109,110^. There is substantial evidence suggesting sex differences in prevalence and efficiency of CB1 receptors, metabolism of cannabis, and behavioral responses^111,112^. To incorporate this knowledge and protect participants, future studies investigating sex-differences should administer a proportional dose based on weight to avoid attrition, as five of the studies did^46,53,79,81,82,87^. Evidence regarding sex-effects are mixed, with some results indicating long-term behavioral changes may be greater for males than females, illustrating the need for more in-depth studies adequately powered to examine sex-differences^113^.

### Overlapping studies

Several of the reviewed studies reported results from different tasks acquired from the same experiment, which is important to acknowledge as discussing them independently inflates sample of participants in the literature. Studies that reported on the same data set are indicated in Table 1 with matching asterisks. Additionally, ten of the studies did not indicate that they drew from overlapping samples; however, the demographic summary statistics of participants indicate that they likely are^38,43,44,67–71,74,86^. It is vital to weigh interpretations of these findings with knowledge that there may be limitations to generalizability and bias due to the subjects recruited possibly leading to inflated estimates of statistical significance^114^. Among the significant results from these studies are the opposition of THC and CBD, limiting the generalizability of the results. Judging purely by the number of papers published, the casual reader may obtain an inflated perspective on the number of neuroimaging cannabis studies. While they provide a strong foundation, the limited number of unique participants (~733), and the homogeneity of the samples greatly compromises the generalizability of results.

## Conclusion

While the effects of cannabis exposure have become a focal point for research in recent years, much remains unknown despite the rapid legalization of cannabis around the world. This paper fills an important gap by providing a systematic review of studies that administer THC, not only suggesting potential effects of acute THC exposure but also drawing attention to certain limitations confronted by the field as a whole. Future work should consider researching long-term cannabis exposure in rodents, characterizations of dose-response relationships, sex-differences in sensitivity, and differences across mechanisms of exposure, such as oral consumption versus inhalation. A deeper understanding of the potential harms and benefits of cannabis exposure in humans requires a multifaceted examination of the effects on neurodevelopment.

## Appendix

Bossong, Matthijs G., et al. Further human evidence for striatal dopamine release induced by administration of∆ 9-tetrahydrocannabinol (THC): selectivity to limbic striatum. *Psychopharmacology* 232.15 (2015): 2723–2729. **Excluded because evidence synthesized two included papers.**

Freeman, T. P., Pope, R. A., Wall, M. B., Bisby, J. A., Luijten, M., Hindocha, C., ... & Morgan, C. J. (2018). Cannabis dampens the effects of music in brain regions sensitive to reward and emotion. *International Journal of Neuropsychopharmacology*, 21(1), 21–32. **Excluded because sample fell outside of the age-range included.**

Bedi, Gillinder, Martin A. Lindquist, and Margaret Haney. An fMRI-based neural signature of decisions to smoke cannabis. *Neuropsychopharmacology* 40.12 (2015): 2657–2665. **Excluded because sample fell outside of the age-range included.**

O'Leary, D. S., et al. Acute marijuana effects on rCBF and cognition: a PET study. *Neuroreport* 11.17 (2000): 3835–3840. E**xcluded because sample fell outside of the age-range included.**

van Hell, Hendrika H., et al. Methods of the pharmacological imaging of the cannabinoid system (PhICS) study: towards understanding the role of the brain endocannabinoid system in human cognition. *International Journal of Methods in Psychiatric Research* 20.1 (2011): 10–27. **Excluded because description of methodology without report of results (included in other papers).**
